# Interference with Locomotion as a Uterine Symptom

**Published:** 1887-08

**Authors:** 


### INTERFERENCE WITH LOCOMOTION AS A UTERINE SYMPTOM.

GRAILY HEWITT, M.D., F.R.C.P., of London, says: Considering the very large number of cases in which "inability to walk easily," "pain on walking," or various modifications of the symptom occur, it is remarkable that so few practitioners, comparatively speaking, pay any attention to it, or even treat the symptom seriously. There can be no doubt, however, that patients themselves look upon this symptom as a serious one, and indeed in most cases speak of it, when they are allowed to express their unbiased opinion on the subject, as an inconvenience which most seriously affects their comfort in life, and for relief of which they would be willing to make great sacrifices.

The above is a general statement. Any amount of evidence is obtainable in regard to its accuracy by interrogating patients, which should be done, not by leading questions, but by requesting them to state what are the principal inconveniences of which they complain. This method of inquiry will reveal the fact, that patients attach more importance to this inability for locomotion as interfering with their comfort than almost any other. For this reason, if for no other, this symptom is deserving of particular attention, and its explanation is a matter of great interest.

There is no difference of opinion as to the necessity for rest and the horizontal position in cases where there is found to be present marked tenderness or signs of inflammatory action in the uterus or its immediate neighborhood, the presence of such inflammatory action being revealed more or less decidedly by the results of a digital examination. It is not necessary to point out in such cases as these that difficulty or pain in locomotion are connected with, and due to, the inflammatory condition of the uterus or structures adjacent. In such cases no one considers it surprising that the patients should prefer to keep quiet, and the medical attendant usually and very properly advises that the horizontal position should be maintained.

There is another class of cases in which the pain on movement is found to be connected with laceration of the cervix and os uteri during some previous labor. These cases present themselves oftener than is generally supposed; the torn surface is continually irritated and stretched when the patient is walking, or standing, eversion of the lining of the cervix occurs, and a chronic source of irritation is set up. It is not infrequent in such cases to find also, from time to time, small localized cellulitic exudations and more or less tenderness to the touch.

The cases I have however more particularly in view, are those in which no particular sign or presence of inflammatory lesion of the body of the uterus or of the cervix and os uteri is detected on vaginal examination, no tenderness to touch, and an absence indeed of what may be termed spontaneous pain. The characteristic is, that so long as the patient remains quiet, there may be little discomfort; but the upright position, or the act of walking, induces pain or an uncomfortable sensation, which is so decided, that in the end the patient avoids walking as much as possible. Thus originates in not a few cases a chronic invalidism, the starting point of which will, on inquiry, generally be found to have been habitual pain on attempting ordinary exertion.

Patients suffering in the manner now described, and not presenting obvious signs of presence of inflammatory action in the pelvis, are often looked on as fanciful, or hysterical, or disposed to exaggerate their sufferings. No doubt there are differences in regard to the manner in which different individuals bear pain, but the pain nevertheless has to be accounted for, and in the most typical cases it must be remarked, the patient is obviously and strongly desirous of living an active life, and anxiously seeks means to enable her to do so.

There are, it should be here remarked, cases in which general debility, associated with disorder of the heart or lungs, or general nutrition, giving rise to a certain incapacity for locomotion; but in such cases what the patient complains of is prostration after walking, whereas in the cases to which I desire to call attention, the walking power is often really good, but the exercise of that power gives rise to such discomfort internally that it cannot be kept up for long together.

Associated with the presence of pain in one or other groin, or in the back, there is frequently experienced a bearing-down sensation. The most common seat of this pain is in the groin or the pelvis on one side or other behind this spot. Now, the question is, what interpretation is to be placed on the pain arising from locomotion, when not obviously connected with some inflammatory condition ? The answer is that the movement in question is painful because it imparts an unusual amount of movement, or an unusual kind of movement, to the uterus or to certain parts of the uterus. The uterus enjoys naturally a power of motion backwards, forwards, laterally, and upwards or downwards, within certain restricted limits. It enjoys flexibility also within certain limits. Unusual flexibility is not seldom associated with excess in degree of the other movements, as well as with peculiar tendency to lateral movements.

These excessive movements are, or may be, painful and productive of pain. They are liable to be produced by standing or by locomotion. Frequently they are brought about in the first instance by excessive locomotion or analogous causes (straining, over-exertion, etc.), and subsequently the evil state of things thus produced is intensified and temporarily aggravated by even trifling exertion.

The proof that the foregoing is the true explanation is easily obtainable by watching a few cases, and observing the effects produced on the position and shape of the uterus by exertion. Not less useful in obtaining valuable evidence is the analysis of the patient's symptoms, as described by herself, whereby it is made often quite clear how and why it is that the discomfort complained of arises.

In most cases where this kind of pain is experienced, the uterus falls lower than usual in the pelvis, but more generally we find that the body of the uterus is the part which particularly is to be felt lower than usual. In fact, according to my experience, simple descent of the uterus, unattended by marked version or flexion, gives rise to little inconvenience within certain limits. As regards retroflexion, most authorities are content to regard this as a real pathological condition. As regards anteflexion, however, which I believe is responsible for the pain now under discussion, in the large majority of cases, there is less unanimity. In fact, many deny that anteversion and anteflection are pathological at all. True, slight anteflexion and slight anteversion may be admitted as a definition of the shape and position of the normal uterus, but there are various degrees of this ante-displacement and flexion met with, which as the result of my experience are liable to be attended with grave symptoms. The pain described above as produced by locomotion, is one of the grave symptoms produced by severe anteversion or anteflection of the uterus. The reason that this has not been generally admitted is, I believe, that sufficient pains have not been taken to differentiate slight normal anteversion and anteflexion with a properly mobile uterus from severe degrees of this kind of displacement and change of shape. The normal anteflexed uterus oscillates within slight range, recovering its position and shape after moderate disturbance. The abnormally flexible uterus gives way to a greater extent, and the extra amount of flexion, as well as descent involved, gives rise to pain. In cases where the uterus has gradually fallen lower and lower it is apt to become fixed, and does not recover its true position after exertion. This constitutes a condition in which fresh exertions occasion pain, owing to the increased pressure downwards, and under these circumstances, the slightest exertion gives rise to discomfort.

"Uterine dyskinesia" is the term which I have employed in describing the symptoms specially defined in the foregoing remarks. It is as common as leucorrhœa, and more common than most other uterine symptoms, and has a very definite and well-marked place in uterine symptomatology.

One of the most important arguments adduced in reference to "uterine dyskinesia," its nature and explanation, is that whatever tends to preserve the uterus in a state of repose is almost invariably successful in giving the patient relief. In acute cases of flexion, even of the worst kind, great benefit is derived from placing the patient in such a position that the force of gravity no longer acts unfavorably upon the uterus. Thus, in cases of anteflexion the recumbent dorsal position with hips raised is the one to be selected, whereas in cases of retroflexion the very opposite is necessary. Still more marked results follow when steps are taken by internal mechanical treatment, to raise the fundus, whereby the flexion is lessened in a more decided manner. Now, the fact that these beneficial results do follow, and the patients obtain—it may be said almost invariably—relief by these positional or mechanical methods of treatment, constitutes an argument of no little value in favor of the view of the subject which has been above maintained.

In the foregoing observation I have made no attempt to show why it is that excessive movements and change of shape of the uterus therewith associated, give rise to pain during locomotion. I have simply insisted on the fact that difficulty in locomotion in women is so very generally met with in cases where these excessive movements and changes of shape of the uterus exist, that there can be no doubt that there is an intimate relation between them, the latter as cause, the former as the effect.—*Quarterly Compendium of Medical Science.*

